# Quantifying Root Resorption on the Incisors After Clear Aligner and Fixed Appliance Therapy Using Artificial Intelligence Tool Based CBCT Surface Models: Randomized Clinical Trial

**DOI:** 10.1111/ocr.70082

**Published:** 2025-12-30

**Authors:** Roberto Bespalez‐Neto, Claudia Trindade Mattos, Lucia Helena Soares Cevidanes, Thais Maria Freire Fernandes, Ana Cláudia de Castro Ferreira Conti, Renata Rodrigues de Almeida‐Pedrin, Paula Vanessa Pedron Oltramari

**Affiliations:** ^1^ Department of Orthodontics, School of Dentistry Uniderp Campo Grande Mato Grosso do Sul Brazil; ^2^ Department of Orthodontics Universidade Federal Fluminense Rio de Janeiro Brazil; ^3^ Department of Orthodontics and Paediatric Dentistry, School of Dentistry University of Michigan Ann Arbor Michigan USA

**Keywords:** class I malocclusion, clear aligner, external apical root resorption, fixed appliances, orthodontics

## Abstract

**Objective:**

To quantify external apical root resorption (EARR) on the incisors following non‐extraction treatment of Class I malocclusion patients with moderate crowding, comparing clear aligners and fixed appliances using a novel 3D analysis of Cone‐Beam Computed Tomography (CBCT) derived surface models.

**Methods:**

In this randomized clinical trial, 32 adult patients, mean age 22.3 years, mean treatment duration 24.2 months, with Class I malocclusion and moderate crowding (mean Little's Index 4.76 mm) were allocated to either clear aligner (*n* = 15) or fixed appliance (*n* = 17) treatment. CBCT scans were obtained before and after treatment. EARR was measured using surface‐based analysis of 3D models, and associations with patient and treatment‐related factors were tested.

**Results:**

The overall median EARR was −0.72 mm, with no significant difference between clear aligners (−0.71 mm) and fixed appliances (−0.72 mm). Upper lateral incisors exhibited significantly greater EARR than lower incisors (*p* = 0.002) and upper central incisors (*p* < 0.001). No significant predictor for EARR was found considering age, sex, crowding severity or treatment duration.

**Conclusions:**

EARR occurred following non‐extraction treatment of Class I malocclusion with both clear aligners and fixed appliances, with no significant difference between appliance types. Upper lateral incisors were most susceptible to EARR. The novel 3D analysis enabled comprehensive quantification of total EARR, setting a new methodological standard. Monitoring root health during treatment is important, particularly for upper lateral incisors.

## 1 Introduction

External apical root resorption (EARR) represents a significant complication of orthodontic treatment that can compromise periodontal health and tooth longevity [1, 2]. The amount of EARR varies depending on patient‐related factors such as genetics [3] and root morphology and treatment‐related factors, particularly the magnitude and direction of orthodontic forces [4, 5], amount of orthodontic movement and treatment duration [6]. While any tooth can be affected, EARR is most evident on the maxillary and mandibular incisors, with an average reduction in root length reported to be less than 1.5 mm [7, 8, 9, 10, 11, 12]. Accurate diagnosis and monitoring of EARR during treatment is essential for ensuring optimal outcomes and long‐term stability [1].

Traditionally, EARR has been assessed using periapical radiographs, [12, 13] panoramic radiographs [14, 15, 16] and more recently, cone‐beam computed tomography (CBCT) [7, 9, 10, 11]. However, radiographic methods are limited by their two‐dimensional nature, with periapical and panoramic images prone to distortion from changes in tooth angulation [17]. While CBCT provides three‐dimensional visualisation, most studies have relied on linear measurements on 2D slices, [9, 11, 17, 18, 19, 20, 21] which may not fully capture the complex volumetric changes in root morphology.

To date, comparisons of EARR between clear aligner therapy and fixed appliances have yielded inconsistent results, with some studies reporting less EARR with aligners [20, 21, 22, 23, 24, 25] and others finding no significant difference [12, 26, 27, 28]. These discrepancies may stem from heterogeneity in sample characteristics and treatment protocols, with different malocclusions and extraction patterns potentially influencing the amount of tooth movement and associated EARR. Furthermore, the relationships between EARR and patient factors such as age [9, 18, 25] and sex [9, 19, 25] and treatment factors like crowding severity and duration remain unclear due to conflicting findings in the literature [9, 18, 19, 26, 29].

Therefore, the primary objective of this study was to quantify the amount of EARR following non‐extraction treatment of Class I malocclusion with moderate crowding, comparing clear aligners and fixed appliances. To improve upon the limitations of prior studies, we employed a novel methodology based on surface correspondence between initial and final CBCT‐derived 3D models, enabling comprehensive visualisation and measurement of volumetric root changes. Our secondary aim was to explore associations between EARR and patient and treatment‐related variables, leveraging the homogeneous nature of our sample to minimise confounding effects.

## 2 Materials and Methods

This study was a parallel randomised controlled clinical trial wherein participants were prospectively recruited and randomised into two groups. There were no changes in the methods after commencement of the trial. The study followed the Consolidated Standards of Reporting Trials guidelines (CONSORT) [30].

The study was conducted in the Department of Orthodontics of the University of North Parana and was approved by the ethical committee University of North Parana (UNOPAR) (12088219.0.0000.0108) and registered in the Brazilian Clinical Trials (RBR‐9zytwf) registry. Sample size was calculated based on a study by Eissa et al. [31], using the following formula [32]:

$$n=\sigma^{2}\times {\left(Z_{1-\alpha /2}+Z_{1-\beta }\right)}^{2}/d^{2}$$

$$n={\left(120\right)}^{2}\times {\left(1.96+0.84\right)}^{2}/{\left(100\right)}^{2}$$

n=12

The sample size calculation showed that a minimum of 12 patients in each group would be required. With an *alpha* error of 0.05% and 80% power. Considering possible losses to follow‐up, a minimum of 15 patients were collected in each group.

The sample was obtained by the screening of 2662 individuals on social media or in schools in the city of Londrina, Brazil. The inclusion criteria were as follows: Angle Class I malocclusion, moderate crowding (Little Index 4–6 mm), facial symmetry, passive lip seal and nonextraction treatment. The exclusion criteria were as follows: absence of permanent teeth, anterior and/or posterior open bite and/or crossbite, anterior teeth in need of restorations, history of trauma to the maxillary incisors, previous orthodontic treatment and presence of previously occurred EARR.

Simple randomization [33] in a 1:1 ratio was performed by an external researcher using the Excel 2007 program (Microsoft, Redmond, WA, USA). The randomization codes were consecutively inserted in opaque, sealed, numbered envelopes; this ensured concealment of the group allocation. The patients were divided into two groups: group 1, Orthodontic Aligners (OA): 15 patients (7 female, 8 male) treated with orthodontic aligners (SmartTrack, Invisalign; Align Technology, San Jose, CA, USA) and group 2, Fixed Appliance (FA): 17 patients (7 female, 10 male) treated with fixed metallic orthodontic appliance (slot 0.022″ × 0.030″, 3 M Unitek, Monrovia, CA, USA). Each group was divided into three subgroups, lower incisors (LI) (*n* = 128), upper central incisors (U1) (*n* = 64) and upper lateral incisors (U2) (*n =* 64). Therefore, all 256 incisors were analysed.

In group 1 (OA), patients were treated according to the manufacturer's recommendations regarding appliance wear (22 h per day), hygiene and storage. Individualised virtual treatment planning was performed for each patient after evaluation of diagnostic records using ClinCheck Pro software (version 5.6; Align Technology, San Jose, CA, USA), which includes 3D visualisation tools. All system features were used according to the specific clinical needs of each patient. The sequence of clinical procedures followed the virtual treatment plan and included the placement of attachments, interproximal enamel reduction (IPR) and use of intermaxillary elastics, among others. Aligner changes were prescribed every 10 days. The mean amount of interproximal enamel reduction performed was 0.48 ± 0.67 mm in the maxillary arch and 1.49 ± 1.18 mm in the mandibular arch. In group 2 (FA), patients were treated with conventional fixed appliances bonded to all teeth, following the same archwire sequence: nickel‐titanium (0.014″, 0.016″, 0.016 × 0.022″, 0.019 × 0.025″) and stainless steel (0.019″ × 0.025″). All mechanical auxiliaries were applied as needed to address individual treatment requirements. The sequence of clinical procedures followed the initial treatment plan, which included bonding of attachments, interproximal enamel reduction (IPR) and use of intermaxillary elastics, among others. The mean amount of interproximal enamel reduction performed was 0.18 ± 0.67 mm in the maxillary arch and 0.05 ± 0.16 mm in the mandibular arch.

**FIGURE 1:**
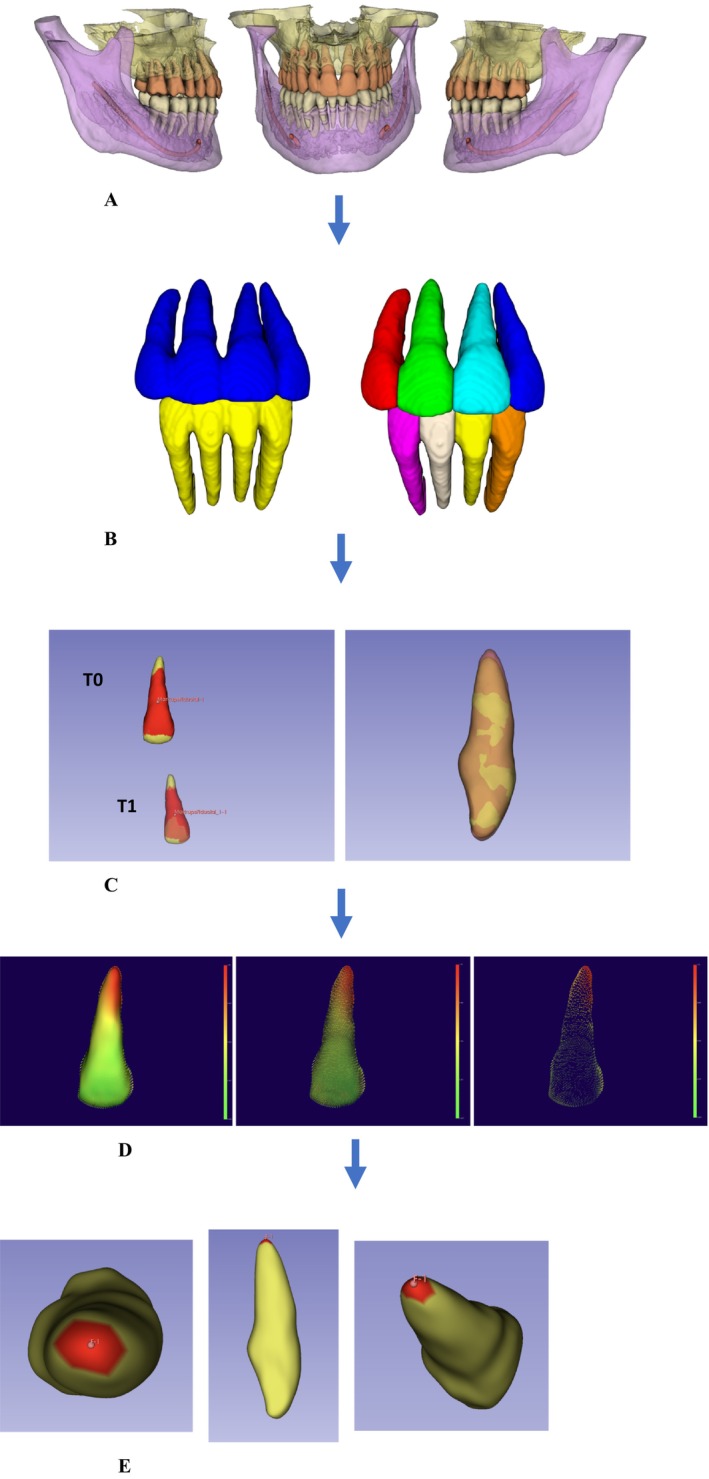
(A) Maxillary, mandibular, upper teeth, lower teeth and mandibular canal segmentation. (B) Individual labelling of incisors. (C) Surface registration of the two timepoints of an upper incisor for superposition. (D) Calculation of models based on corresponding points and the point‐to‐point distance between the two loaded models. (E) Demarcation of the region of interest (ROI) to quantify root resorption.

The treatment was considered finished when the six keys to normal occlusion were reached. From this, the total treatment time was calculated. CBCT scans were performed with CBCT machine *i*‐CAT Next Generation (Imaging Sciences International, Hatfield, PA); voxel size 0.3 mm with FOV 17 cm (H) × 23 cm (D). CBCT scans were obtained at the beginning of treatment (T0) and at the end of active treatment (T1). The Figure 1 shows the applied methodology sequence. The images were segmented in the 3D Slicer (https://www.slicer.org version 5.7.0) software using an Artificial Intelligence (AI) based tool, Dental Segmentator [34], which automatically divides the volume into specific labelled parts, maxilla and base of the skull, mandible, mandibular canal, upper teeth and lower teeth (Figure 1A). The segmented file was imported into the ITK‐Snap (http://www.itksnap.org version 4.4.0) software, where the volume of the mandibular canal, mandible and skull base were excluded from the image. Also, in ITK‐Snap, the teeth, canines, premolars and molars were excluded and the upper and lower incisors were individually separated (Figure 1B). After this step, the entire sample was categorised into lower incisors (LI), upper central incisors (U1) and upper lateral incisors (U2), since they are homologous teeth and have the same root morphology. Using the CMF Registration tool (3D Slicer), the tooth files at T0 and T1 were superimposed for comparison between timepoints. To avoid superimpose errors, in both models (T0 and T1), the area close to the cementoenamel junction was selected, excluding the root apex and incisal edge, as these are the places where the surface changes occur (Figure 1C). Next, the SPHARM [35] was used to generate a mesh approximation from the segmented label files with over 4000 points per tooth, compute the parameterization of corresponding surface points among the incisors, calculate the models based on corresponding points and then the point‐to‐point distance between the two loaded models was calculated (Figure 1D). To quantify root resorption, the Region of Interest (ROI) was demarcated (Figure 1E) and the measurements were automatically calculated by the Mesh Statistics tool between the points of the two models on the demarcated area. The data was exported with the maximum (largest negative value), 5 percentile (corresponding to the 95% percentile as the values of resorption are negative) and 25 percentile (corresponding to the 75% percentile) measurements and tabulated for statistical processing. The maximum value corresponded to the point where the greatest resorption was observed within the ROI; the 5‐percentile corresponded to a threshold value that excludes potential outliers, serving as a conservative maximum and the 25 percentile corresponded to the value below which 75% of the resorption measurements within the ROI fell, providing insight into the distribution of more severe resorption points.

### 2.1 Statistical Analysis

Operator blinding was not possible. However, a blind external researcher statistically analysed the collected data. The selected CBCT scans were anonymized one by one using the Dolphin Imaging software version 11.95 (Patterson Dental, Chatsworth, CA) anonymization tool and coded G1 and G2 for group 1 and group 2 respectively and P1 to P17 for the patient number. Statistical analysis was done using JAMOVI (macOs version 2.5.7.0, Sydney, Australia) and Numbers software (version 2.02010–2024 Apple Inc., Strobe Inc.). The data were checked for normal distribution by using the Shapiro–Wilks test. Age, Little's index and treatment duration were considered as presenting normal distribution. Resorption differences were considered as having irregular distribution; therefore, nonparametric tests were used and median and interquartile ranges displayed. To evaluate intra‐examiner error, 30% of the measurements were repeated, including the surface registration method and the results evaluated using the intraclass correlation coefficient (ICC) and Bland and Altman agreement tests, following the criteria described by Fleiss [36]. Intergroup comparisons were performed using independent *t* tests (age, Little's index and treatment duration) and the Chi‐square test (sex). Intergroup comparisons of EARR were performed using Mann–Whitney tests. Intragroup comparisons of one sample were tested with Wilcoxon rank to assess their difference to the absolute value of 0. *p* < 0.05 was taken as the significance level. A linear regression was performed to test the influence of predictors group, age, sex, Little's index, treatment duration and type of teeth on the amount or EARR.

## 3 Results

**FIGURE 2:**
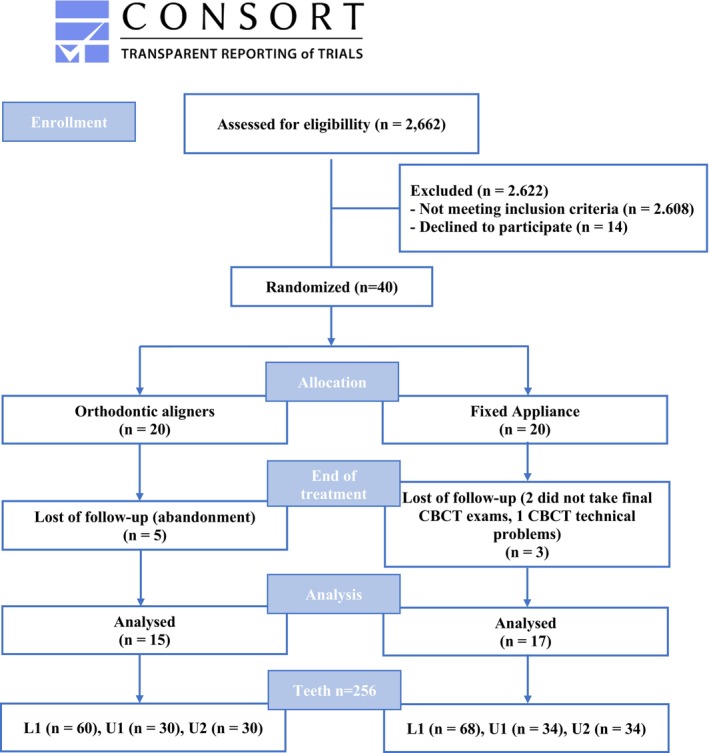
Consolidated Standards of Reporting Trials (CONSORT) diagram showing the flow of patients through the trial.

Figure 2 shows the flowchart of patients assessed for eligibility, randomization, allocation, treatment, and monitoring end of treatment. Participants who fulfilled the established criteria were recruited between August 2018 and February 2019. Of the 2662 individuals screened, a total of 54 patients met the criteria, although only 40 showed interest in receiving treatment. Orthodontic examinations were performed in February 2019. In May 2019, patients came for the post randomization (baseline) appointment, appliance installation and instructions. In the clear aligner group, five patients abandoned treatment, while in the fixed appliance group, two patients refused to undergo CBCT final scans and one patient was not analyzed due to technical problems in the CBCT. The mean Little's index was 4.54 mm (SD 1.33) for the orthodontic aligners (OA) group and 4.95 mm (SD 1.87) for the fixed appliance (FA) group. The mean treatment duration was 26.2 months (SD 7.84) for the OA group and 22.4 months (SD 6.07) for the FA group. No statistically significant difference was found between the groups. The mean number of refinements in the OA group was 2 (SD 0.8). Age, Little's Irregularity Index and treatment duration are summarized in Table 1.

**TABLE 1:** Characteristics of the sample and treatment divided by groups.

	Group 1 OA (*n* = 15)	Group 2 FA (*n* = 17)	
Variable	Mean (SD)	Mean (SD)	*p*
Age (year)	24.1 (6.30)	20.7 (4.79)	0.099
Little's index (mm)	4.54 (1.33)	4.95 (1.87)	0.491
Treatment duration (month)	26.2 (7.84)	22.4 (6.07)	0.135

According to Fleiss [36], the result of intra‐examiner error indicated good to excellent reproducibility, confirmed by the intraclass correlation coefficient (ICC) of 0.808. The Bland–Altman analysis showed a bias estimate of −0.048 (95% confidence interval, −0.114, 0.018), with limits of agreement of −0.597 and 0.501. That indicated minimal systematic bias and small magnitude of error between the repeated measures.

Radicular resorption was detected in both groups and all teeth evaluated showed a reduction in root length, significantly different from zero. The overall median resorption, considering lower and upper incisors of the sample, was −0.72 mm (IQR 0.42). When evaluating the type of tooth, the median resorption observed was: LI lower incisors −0.73 mm (IQR 0.39), U1 upper central incisors −0.64 mm (IQR 0.34) and U2 upper lateral incisors −0.80 mm (IQR 0.55) (Table 2).

**TABLE 2:** Median EARR of the entire sample and divided by teeth type and *p*‐value of the Wilcoxon rank one‐sample test comparing the medians to the value of zero.

Descriptives	*n*	Median (mm)	IQR	*p*
Overall	256	−0.72	0.42	< 0.001
LI	128	−0.73	0.39	< 0.001
U1	64	−0.64	0.34	0.002
U2	64	−0.80	0.55	< 0.001

*Note:* Statistically significant difference (*p* < 0.05).

Abbreviations: IQR, Interquartile Range; LI, Lower Incisors; U1, Upper Central Incisors; U2, Upper Lateral Incisors.

Intergroup evaluation showed maximum resorption for group 1 (orthodontic aligner OA): LI lower incisors −0.74 mm (IQR 0.29), U1 upper central incisors −0.59 mm (IQR 0.19) and U2 upper lateral incisors −0.80 mm (IQR 0.59) and group 2 (fixed appliance FA): LI lower incisors −0.70 mm (IQR 0.47), U1 upper central incisors −0.69 mm (IQR 0.53) and U2 upper lateral incisors −0.79 mm (IQR 0.41). No statistical difference was observed when comparing groups (Table 3).

**TABLE 3:** Comparison of the differences in correspondence surface from T0 (before treatment initiation) to T1 (end of active treatment), which represents the amount of external apical root resorption between the orthodontic aligner (OA) and fixed appliance (FA) groups.

		Group 1 OA	Group 2 FA	
Reference	Type of tooth	Median (IQR)	Median (IQR)	*p*
Maximum	LI	−0.74 (0.29)	−0.70 (0.47)	0.562
	U1	−0.59 (0.19)	−0.69 (0.53)	0.435
	U2	−0.80 (0.59)	−0.79 (0.41)	0.733
Per 5	LI	−0.72 (0.27)	−0.67 (0.45)	0.507
	U1	−0.57 (0.20)	−0.65 (0.49)	0.543
	U2	−0.77 (0.55)	−0.76 (0.41)	0.626
Per 25	LI	−0.60 (0.30)	−0.58 (0.39)	0.507
	U1	−0.51 (0.16)	−0.61 (0.52)	0.877
	U2	−0.74 (0.54)	−0.69 (0.40)	0.552

*Note: p*‐values were calculated using the U Mann–Whitney test. Statistically significant difference (*p* < 0.05).

Abbreviations: IQR, Interquartile Range; LI, Lower Incisors; Per 5–Per 25, Percentile; U1, Upper Central Incisors; U2, Upper Lateral Incisors.

The result of the linear regression is shown in Table 4. The influence of group type (OA or FA) in the amount of EARR was not statistically significant (*p* = 0.693). No statistically significant influence of age (*p* = 0.524), sex (*p* = 0.102), degree of initial crowding measured by Little's index (*p* =0.845) and, duration of active orthodontic treatment (*p* = 0.144) was detected. However, when the type of teeth made a significant impact on the amount of EARR, differently between upper lateral incisors (U2) and lower incisors (LI) (*p* = 0.002) and between upper lateral incisors (U2) and upper central incisors (U1). The upper lateral incisors had the greater mean amount of EARR, followed by lower incisors and upper central incisors.

**TABLE 4:** Linear Regression analysis considering group, age, sex, Little's index, treatment duration and type of teeth as independent variables.

Predictors	Estimates	Standard error	*p*
Intercept^a^	−0.71703	0.15038	< 0.001
Group: 2–1 (reference group 1 OA)	−0.01933	0.04898	0.693
Age	−0.00276	0.00432	0.524
Sex: M–F (reference female)	−0.07569	0.04616	0.102
Little's index	0.00281	0.01439	0.845
Treatment duration	−0.00504	0.00344	0.144
Teeth: LI‐U2 (reference U2)	0.17737	0.05521	0.002
Teeth: U1‐U2 (reference U2)	0.24639	0.06375	< 0.001

Model	*r*	*r* ^2^	
1	0.283	0.0801	

*Note:* Models estimated using sample size of *n* = 256. Statistically significant difference (*p* < 0.05).

Abbreviations: LI, Lower Incisors; U1, Upper Central Incisors; U2, Upper Lateral Incisors.

^a^ Represents the reference level.

## 4 Discussion

This randomized clinical trial quantified external apical root resorption (EARR) following non‐extraction treatment of Class I malocclusion patients with moderate crowding, comparing clear aligners and fixed appliances using a novel 3D analysis of CBCT‐derived surface models. Our results revealed an overall median EARR of −0.72 mm, with no significant difference between appliance types. Upper lateral incisors exhibited greater EARR than lower incisors and upper central incisors. Age, sex, crowding severity and treatment duration were not significant predictors of EARR.

Patient compliance with aligner wear was assessed using several complementary methods to ensure comprehensive monitoring throughout treatment. Self‐reported questionnaires related to aligner use were administered as part of an ongoing study, with results indicating that 55% of patients reported wearing aligners ≥ 20 h/day, 20% reported 15–20 h/day, and 20% reported 10–14 h/day, which was considered acceptable for a removable appliance modality. Monthly follow‐up visits were conducted during which patients brought their used aligners for evaluation of compliance indicators; specifically blue wear‐time markers that become transparent when aligners are worn for the recommended duration. Additionally, periodic intraoral scans were performed to monitor treatment progress using the Progress Assessment tool (OrthoCAD), verifying whether clinical outcomes matched the virtual treatment plan. Finally, a validated Cooperation Instrument [37] was administered by a single professional who followed all patients throughout treatment. Results from this instrument showed no significant difference in cooperation between groups during the first year of treatment—the period for which the instrument is validated—as previously published [38].

The incidence of EARR in our sample aligns with previous studies reporting average resorption between 0.47 [19] and 1.69 mm [8], with most finding less than 2 mm of EARR [7, 8, 9, 10, 12, 29], although other studies have not used the same methodology as the current study. This consistency across diverse populations and methodologies underscores the pervasiveness of EARR as an undesirable yet common consequence of orthodontic tooth movement.

A key finding was the lack of significant difference in EARR between clear aligners (−0.71 mm) and fixed appliances (−0.72 mm), corroborating prior comparisons [12, 26, 27, 28]. This challenges the notion that aligners are inherently less damaging to roots. While some studies have reported less EARR with aligners [20, 21, 22, 23, 24, 25], this discrepancy likely reflects heterogeneity in sample characteristics and treatment protocols. Notably, the amount of EARR is directly related to the extent of tooth movement and the magnitude of applied forces [2, 5]. Thus, studies comparing treatment of different malocclusions or using varying treatment strategies may be confounded by differences in biomechanical factors. By focusing on a well‐defined malocclusion type, a similar amount of crowding and non‐extraction approach, our study minimised such confounding effects, providing a more valid comparison of appliances with respect to EARR.

The greater EARR observed in upper lateral incisors relative to lower incisors and upper central incisors is consistent with many previous findings [11, 16], though some studies have reported the opposite pattern [18, 19, 26, 29]. This vulnerability of upper laterals may relate to their smaller root surface area and more frequent engagement in complex tooth movements like torque and rotational corrections. From a clinical perspective, our results suggest that upper laterals warrant extra attention during treatment planning and monitoring for signs of EARR.

Interestingly, we found no significant predictors to EARR among patient factors like age and sex, nor among treatment variables such as crowding severity and duration. While this aligns with some prior investigations, others have reported significant relationships [19, 29]. These inconsistencies likely reflect the complex, multifactorial nature of EARR, with individual susceptibility determined by an interplay of genetic, systemic and local factors [3]. Our study's strict inclusion criteria may have limited the range of these variables, precluding detection of subtle associations. Larger, prospective studies are needed to clarify risk factors for EARR.

A notable strength of our study methodology was the use of surface‐based CBCT analysis to comprehensively visualise and measure root surface changes. Prior CBCT studies have typically relied on linear measurements on 2D slices or non‐corresponding triangulation of surface meshes [9, 11, 17, 18, 19, 20, 21], which may not fully capture the complex morphology of EARR. By leveraging point‐to‐point surface correspondence between 3D models, our approach enabled more anatomically valid quantification of total EARR. This sets a new methodological standard for future research on root resorption.

Certain limitations should be acknowledged. First, our sample was restricted to adults with Class I malocclusion and moderate crowding treated without extractions. While this bolstered internal validity, generalizability to other populations and treatment scenarios may be limited. Second, CBCT scans were obtained only at baseline and end of treatment, precluding analysis of EARR timing and progression. More frequent imaging would be ideal but difficult to justify given the added radiation exposure.

This study provides novel insights into the occurrence and extent of EARR in non‐extraction treatment of Class I malocclusions with moderate crowding. The utilisation of surface‐based CBCT analysis with AI‐based tooth segmentation enabled comprehensive quantification of total EARR, setting a new methodological standard for future research. Our findings underscore the pervasiveness of EARR as a common side effect of orthodontic tooth movement, with no significant difference between clear aligners and fixed appliances. The greater vulnerability of upper lateral incisors highlights the need for vigilant monitoring and proactive management strategies. While there are no predictors to EARR among patient or treatment‐related variables in our sample, larger prospective studies are warranted to further elucidate risk factors. As the field of orthodontics continues to evolve, ongoing research into the mechanisms, prediction and prevention of EARR will be crucial for optimising treatment outcomes and long‐term dental health.

From a clinical perspective, we emphasise the importance of monitoring root health during orthodontic treatment. This is particularly critical for treatments involving aligners, as the movement plan is designed virtually through a digital setup. Special attention must be given to the magnitude of tooth movements between aligners (movement steps) to minimise the risk of significant root resorption.

## 5 Conclusions

This randomized clinical trial demonstrated an overall median external apical root resorption (EARR) of −0.72 mm following non‐extraction treatment of Class I malocclusions with moderate crowding, with no significant differences observed between patients treated with clear aligners and those with fixed appliances. Among the teeth analysed, upper lateral incisors were more prone to EARR compared to lower incisors and upper central incisors. Notably, this study identified no significant predictors of EARR, including age, sex, severity of crowding, or treatment duration within this sample. The application of surface‐based CBCT analysis provided precise and comprehensive visualisation of root surface changes, establishing a robust methodological benchmark for future investigations into EARR and its clinical implications.

## Author Contributions

**Roberto Bespalez‐Neto:** conceptualization, methodology, investigation, writing – original draft. **Claudia Trindade Mattos:** formal analysis, writing – review and editing. **Lucia Helena Soares Cevidanes:** methodology, writing – review and editing. **Thais Maria Freire Fernandes:** writing – review and editing. **Ana Cláudia de Castro Ferreira Conti:** writing – review and editing. **Renata Rodrigues de Almeida‐Pedrin:** writing – review and editing. **Paula Vanessa Pedron Oltramari:** methodology, supervision, writing – review and editing.

## Consent

Informed Consent: The subjects gave informed consent. The manuscript has not been previously published. All authors made a significant contribution to the manuscript and agree to publish data from this research. The protocol for this study received approval from the Human Research Ethics Committee of UNOPAR (CAAE number: 12088219.0.0000.0108).

## Conflicts of Interest

The authors declare no conflicts of interest.

## Data Availability

The data that support the findings of this study are available from the corresponding author upon reasonable request.
